# Intestinal Spirochetosis in an Immunocompetent Patient

**DOI:** 10.7759/cureus.2328

**Published:** 2018-03-15

**Authors:** Patricia Guzman Rojas, Jelena Catania, Jignesh Parikh, Tran C Phung, Glenn Speth

**Affiliations:** 1 Internal Medicine, UCF College of Medicine; 2 Infectious Diseases, Orlando Va Medical Center, UCF Com/hca Gme Consortium's Internal Medicine Residency Program; 3 Pathology, Orlando VA Medical Center; 4 Infectious Diseases, Orlando VA Medical Center; 5 Gastroenterology, Orlando VA Medical Center

**Keywords:** spirochetes, colitis, spirochetosis

## Abstract

Intestinal spirochetosis (IS) is an infestation defined by the presence of spirochetes on the surface of the colonic mucosa. The implicated organisms can be *Brachyspira aalborgi*or *Brachyspira pilosicoli*.

We present the case of a 66-year-old man with a past medical history of diabetes mellitus, hypertension, morbid obesity, and gastroesophageal reflux. The patient was sent to the gastroenterology clinic for a screening colonoscopy due to a prior history of colonic polyps. The patient was completely asymptomatic as he denies any abdominal pain, diarrhea, melena, or hematochezia. A colonoscopy was done showing colitis in the cecum and at the ileocecal valve, for which random biopsies were taken in the terminal ileum, cecum, and ascending colon. The histopathology result was positive for spirochetosis. Due to this finding, the patient was referred to the infectious diseases clinic, where a rapid plasma reagin (RPR) and human immunodeficiency virus (HIV) tests were found to be negative. Since the patient was immunocompetent and asymptomatic, it was decided to monitor and not initiate antibiotic treatment.

Human IS are not related to non-intestinal spirochetes like *Treponema pallidum*. An infection of *T. pallidum* leads to a malignant picture called syphilitic proctitis and appears in the setting of an immunocompromised patient. The treatment of IS is based on the clinical presentation, severity of symptoms, and immune status. The purpose of this case is to emphasize the correct antibiotic indication in patients with IS.

## Introduction

Intestinal spirochetosis (IS) is the presence of spirochetes on the surface of the intestinal mucosa and was first described in 1967 [1]. The implicated organisms can be *Brachyspira aalborgi* or *Brachyspira pilosicoli* [2-4]. *Brachyspira aalborgi* is a non-pathogenic commensal and *Brachyspira pilosicoli* can become an opportunistic pathogen. Spirochetosis can affect up to 5% of healthy people, this prevalence is found to be higher in patients from India or other parts of Asia [5].

## Case presentation

We present the case of a 66-year-old man with a past medical history of diabetes mellitus, hypertension, morbid obesity, and gastroesophageal reflux disease. He was sent to the gastroenterology clinic for a screening colonoscopy due to a personal history of polyps and mild anemia (hemoglobin of 12.6 mg/dL). The patient also had a positive family history of colorectal cancer. He was completely asymptomatic, denying any diarrhea, melena, hematochezia, or increased mucus in stool.

**Figure 1:**
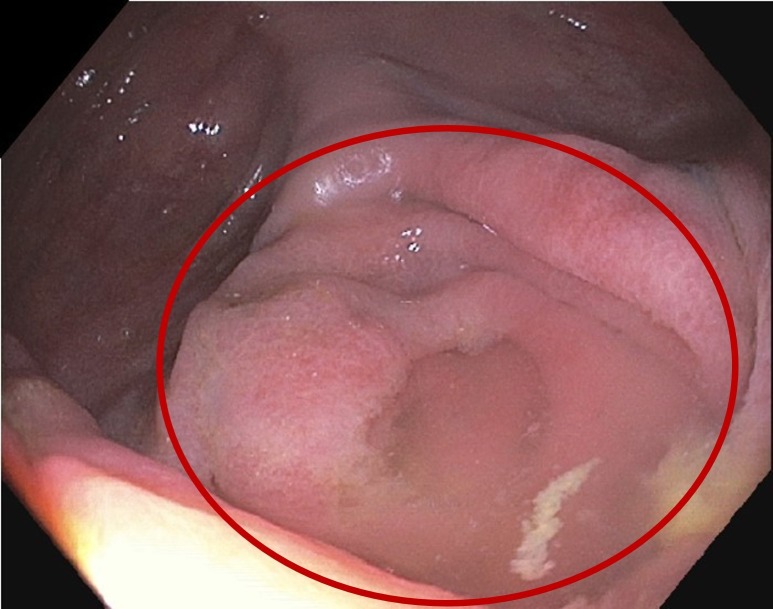
Colonoscopy showing cecum inflammation

**Figure 2:**
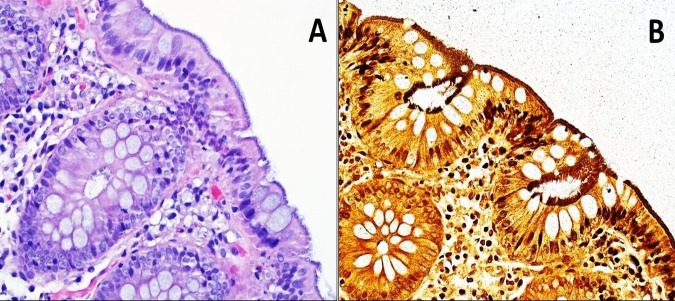
Hematoxylin and eosin (H&E) stain Hematoxylin and eosin stained section of the colonic biopsies showed filamentous structures on the surface epithelium forming a thick bluish fringe (Figure A). A Warthin-Starry silver stain highlighted filamentous organisms (Figure B).

A colonoscopy was done showing severe diverticulosis in the distal descending colon, mid descending colon, and sigmoid colon. There was evidence of colitis in the cecum and at the ileocecal valve for which random biopsies were taken in the terminal ileum, cecum, and ascending colon (Figure 1). The biopsy from the cecum was positive for mild chronic nonspecific inflammation and Warthin-Starry stain was positive for spirochetosis (Figure 2).

Due to the findings, the patient was referred to the infectious diseases (ID) clinic where rapid plasma reagin (RPR) and human immunodeficiency virus (HIV) tests were ordered. Both of them were negative. Since the patient did not have any complaints, the ID clinic decided to monitor the patient and no antibiotic treatment was given.

## Discussion

Spirochetes can be classified into three different families: Spirochaetaceae(*Borrelia, Spirochaeta, Spironema, Treponema*), Leptospiraceae (*Leptonema, Leptospira*), and Brachyspiraceae (*Brachyspira, Serpulina*) [2]. As mentioned above, the two members of the Brachyspiracea family, *B. aalborgi* and *B. pilosicoli*, are associated with IS. Infection with these bacteria follows the fecal-oral route. Furthermore, *B. pilosicoli *is a zoonotic bacterium capable of being transmitted from animals to humans via handling or ingesting meat from infected animals [4].

As in our patient, IS is commonly discovered during a screening colonoscopy in an asymptomatic patient. If the patient becomes symptomatic, he can present with chronic watery diarrhea and/or abdominal pain [6]. Even though IS leads to mild/moderate clinical picture, there are cases where this has been associated with a severe and fatal evolution [2].

Colonoscopy findings are not specific as it can display a polypoid lesion, an erythematous area, or normal mucosa [7]. The diagnosis is made by pathology evaluation of the tissue (the typical histological feature is consistent with a band-like growth of spirochetes, adherent to the colonic luminal surface which is 3-6 um thick). This finding can be seen in the hematoxylin-eosin (H&E) stain; however, Warthin-Starry or Dieterle silver impregnation are stains used for further clarification [2]. An alternative method for diagnosis is the polymerase chain reaction (PCR) test, which targets the 16S rRNA, NDAH-oxidase, and the 23rd DNA gene specific for *B. pilosicoli, B. hyodysenteriae, *and *S. intermedia* [8].

Human IS are not related to non-intestinal spirochetes like *Treponema pallidum* which causes a more malignant picture called syphilitic colitis/proctitis and appears in the setting of an immunocompromised patient. Syphilitic proctitis can cause a tumor-like lesion, hematochezia, tenesmus, or mucous discharge as a clinical picture and mostly affects the rectal area; it begins with an inflammation (endoscopic appearance of erythema, edema, or erosions) that can lead to extensive ulceration. Histologic evaluation can elicit a dense mononuclear cell infiltrate with prominent plasma cells. Granulomas and obliterative endarteritis may be present [9-10].

Treatment of IS must be based on the clinical presentation, severity of symptoms, and immune status. Eradication of symptoms has been reported with metronidazole 500 mg four times a day, for 10 days; however, immunocompetent/asymptomatic patients can be clinically monitored without any initiation of antibiotic treatment [2]. There is no data stating a need for stool studies or repeat colonoscopy.

## Conclusions

The intention of this case is to emphasize the correct antibiotic indication in patients with IS. Moreover, physicians should be aware of the patient’s symptoms and comorbidities/immune status.
